# The Inhibitory Effect of Non-Substrate and Substrate DNA on the Ligation and Self-Adenylylation Reactions Catalyzed by T4 DNA Ligase

**DOI:** 10.1371/journal.pone.0150802

**Published:** 2016-03-08

**Authors:** Robert J. Bauer, Thomas C. Evans, Gregory J. S. Lohman

**Affiliations:** DNA Enzymes Division, New England Biolabs, Inc., Ipswich, MA, 01938-2723, United States of America; Florida International University Bimolecular Sciences Institute, UNITED STATES

## Abstract

DNA ligases are essential both to *in vivo* replication, repair and recombination processes, and *in vitro* molecular biology protocols. Prior characterization of DNA ligases through gel shift assays has shown the presence of a nick site to be essential for tight binding between the enzyme and its dsDNA substrate, with no interaction evident on dsDNA lacking a nick. In the current study, we observed a significant substrate inhibition effect, as well as the inhibition of both the self-adenylylation and nick-sealing steps of T4 DNA ligase by non-nicked, non-substrate dsDNA. Inhibition by non-substrate DNA was dependent only on the total DNA concentration rather than the structure; with 1 μg/mL of 40-mers, 75-mers, or circular plasmid DNA all inhibiting ligation equally. A >15-fold reduction in T4 DNA ligase self-adenylylation rate when in the presence of high non-nicked dsDNA concentrations was observed. Finally, EMSAs were utilized to demonstrate that non-substrate dsDNA can compete with nicked dsDNA substrates for enzyme binding. Based upon these data, we hypothesize the inhibition of T4 DNA ligase by non-nicked dsDNA is direct evidence for a two-step nick-binding mechanism, with an initial, nick-independent, transient dsDNA-binding event preceding a transition to a stable binding complex in the presence of a nick site.

## Introduction

**Fig 1:**
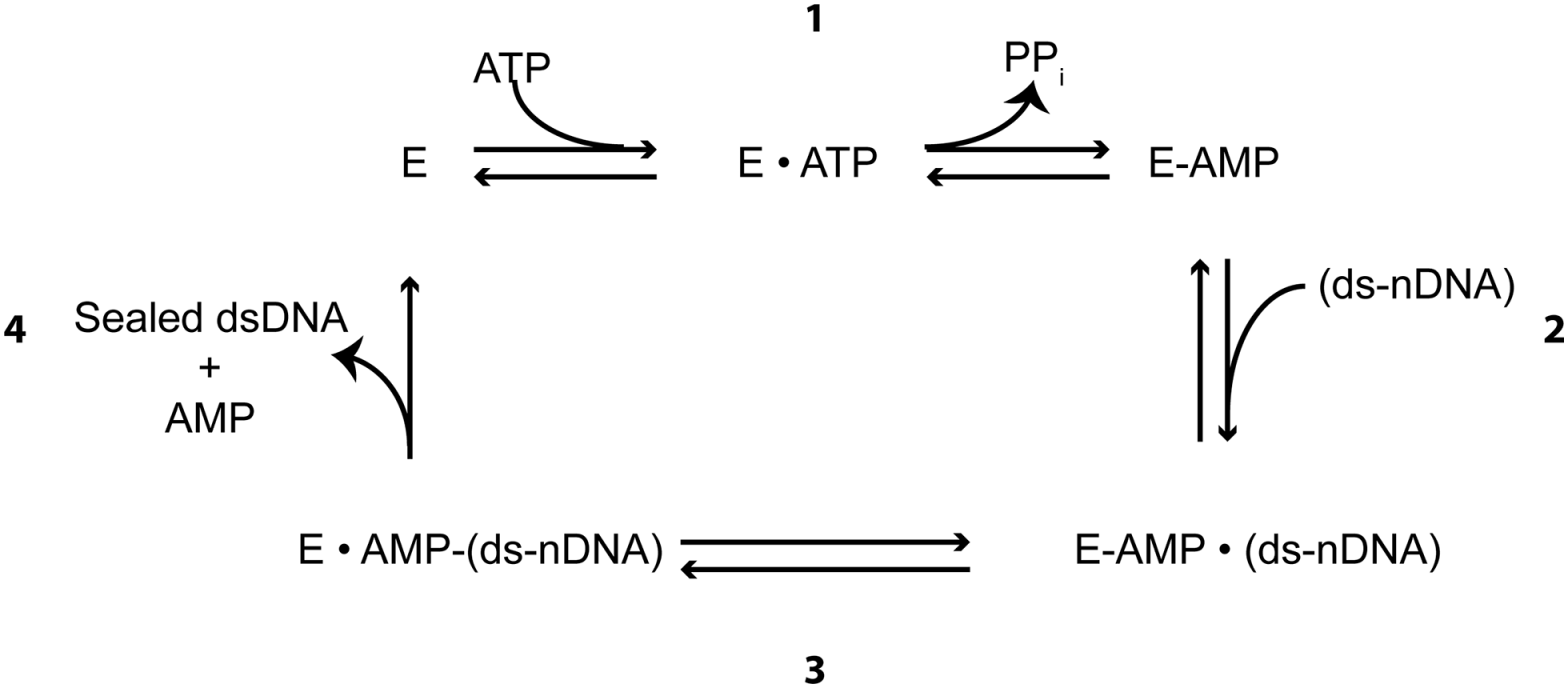
Original Proposed Ligation Reaction Model. The proposed model for nick sealing by a DNA ligase follows three highly conserved nucleotidyl-transfer reactions. **1**. ATP reacts with the ligase active site lysine generating a covalently bound a Lig-AMP enzyme form. **2**. The AMP is transferred from the ligase active site lysine onto the 5’ PO4 of the nick. **3**. AMP is released from the nick upon sealing of the nick by nucleophilic attack from the 3’ OH. Sealing is followed by the release of AMP and sealed dsDNA from the ligase, and then re-adenylylation of the enzyme for subsequent turnover.

DNA ligases are essential enzymes for the *in vivo* maintenance of genome integrity, and are critical to modern *in vitro* biochemical applications. DNA ligases catalyze the formation of a phosphodiester bond between adjacent 3’-hydroxyl and 5’-phosphate termini at the site of a single strand break (“nicked” DNA/ ds-nDNA^1^).[1, 2] Additionally, some ligases can join two dsDNA fragments and substrates with non-adjacent termini.[1–3] DNA ligases are divided into two classes: those dependent on ATP for self-adenylylation, found in eukaryotes, viruses, some bacteria and archaea, and those dependent on NAD^+^, found in bacteria and archaea.[4] In order to perform their nick-sealing function, DNA ligases utilize a ping-pong mechanism involving two substrates and three highly conserved nucleotidyl transfer reactions.[5–9] The pathway begins with the nucleophilic attack by an active site lysine residue on the α-phosphate group of either ATP or NAD^+^, forming an adenylylated ligase intermediate and releasing PP_i_ or β-NMN, respectively (Fig 1, **Step 1**). The adenylylated ligase then binds a 5’-phosphorylated nick site in dsDNA (Fig 1, **Step 2**).[10] After binding, the adenylyl group is transferred onto the 5’ phosphate, producing an AppDNA intermediate and a stably bound ligase-DNA complex (Fig 1, **Step 3**).[10] If a 3’-OH is available, nick closure is achieved through a third nucleophilic attack event by the 3’-OH on the α-phosphate of the AppDNA, resulting in the formation of a phosphodiester bond and release of AMP and the ligated substrate (Fig 1, **Step 4**).[1]

A variety of past studies on DNA ligases have characterized their substrate specificities and enzymatic fidelities.[11–13] The first characterized ligases, from *E*. *coli* and T4 phage, were determined to preferentially bind 5’-phosphorylated DNA.[14–16] This finding has been subsequently reaffirmed in many additional ligases, including those from vaccinia virus, *Paramecium Bursaria* Chlorella Virus (PBCV-1), T7 phage, and human ligases.[11, 17–20] The vaccinia virus and PBCV-1 DNA ligases have also been shown via electrophoretic mobility shift assay (EMSA) to be dependent on the adenylylation state of the ligase as well as presence of a nick for stable complex formation with DNA. Here it was reported that deadenylylated ligase was unable to bind to a phosphorylated nick, and adenylylated ligase was unable to bind to non-nicked dsDNA.[11, 17–19] Two related models have been proposed to explain the DNA ligase nick-sensing mechanism. The first by Rossi *et al*. for T4 DNA ligase postulates nick sensing follows three steps: The first step involves the formation of a transient “T-complex;” where the adenylylated form of the enzyme transiently interacts with dsDNA until it locates a nick site with a 5’ phosphate. In the second step, the enzyme transfers its adenylyl group to the 5’ PO_4_ and forms a stable “S-complex” at the nick site until the DNA is sealed. Upon nick sealing, in the third step, the enzyme is released and immediately re-adenylylated for subsequent rounds of ligation.[10] In the second proposed model, based on crystal structures of *Paramecium Bursaria* Chlorella Virus (PBCV-1) DNA ligase-substrate complexes, nick sensing is facilitated through induction of a 12° bend in the DNA centered on the nick, accompanied by a transition from standard B-form DNA to an A-form like conformation for the two nucleotides at the nick junction site.[21–23] This transition is facilitated by the insertion of the oligonucleotide binding (OB) domain into the DNA minor groove.[24] As dsDNA is less flexible than ds-nDNA, it is more difficult to bend unbroken DNA into the appropriate conformation. Only when the ligase is bound to a nick site can a stable complex with the proper bent DNA conformation be easily achieved. In the case of both of these models, the ligase would require the ability to weakly interact with non-nicked dsDNA as it seeks a nick site. Potential evidence for such an interaction was observed in the active site of the structure of PBCV-1 DNA ligase bound to DNA after the sealing event, where weak contacts between the ligase and its sealed substrate have been described.[21] In further support of this idea are experiments showing AMP-dependent relaxation of supercoiled DNA by T4 and *E*. *coli* DNA ligases, requiring a direct interaction between the deadenylylated ligase and non-nicked dsDNA.[18, 25, 26]

In this manuscript the inhibition of T4 DNA ligase by both non-substrate, non-nicked dsDNA as well as substrate ds-nDNA was investigated. High concentrations of substrate ds-nDNA were shown to result in reduced rates of steady state turnover ligation. It is demonstrated that not only does non-nicked dsDNA inhibit steady state ligation rates for T4 DNA ligase, but that the level of inhibition was proportional to the total dsDNA concentration (per mass volume) present in the reaction, and was independent of dsDNA structure. It is shown that non-nicked dsDNA was able to inhibit the rate of T4 DNA ligase self-adenylylation, implicating an interaction between the deadenylylated form of the enzyme and dsDNA. Finally, through EMSA it is illustrated that non-nicked dsDNA is able to compete with ds-nDNA for binding to T4 DNA ligase. We propose the observed inhibition originates from transient interaction between dsDNA and the ligase, the first direct evidence for the first step in a proposed two-step ligase nick-binding mechanism.

## Materials and Methods

### Materials

*Thermus thermophilus* (*Tth*) DNA ligase (sold under the name *Thermus aquaticus* (*Taq*) DNA ligase by New England Biolabs for historical reasons), PBCV-1 DNA ligase (sold as SplintR ligase by New England Biolabs), T3 DNA ligase, T4 DNA ligase, T7 DNA ligase, 2 M KCl, 1 M MgCl_2_, 1 M DTT, 10 mM ATP, and 50 mM NAD^+^ were obtained from New England Biolabs (Ipswich, MA). Tris-HCl (1 M pH 7.5 @ 25°C) was obtained from Amresco (Solon, OH). Triton X-100 (10%) was obtained from Sigma-Aldrich (St Louis, MO). *Tth* DNA ligase buffer (20 mM Tris-HCl pH 7.5 @ 25°C. 25 mM KCl, 10 mM MgCl_2_, 1 mM NAD^+^, 10 mM DTT, and 0.1% Triton^®^ X-100) was prepared as a 10X stock. T4/PBCV-1 DNA ligase buffer (50 mM Tris-HCl pH 7.5 @ 25°C, 1 mM ATP, 10 mM MgCl_2_, and 10 mM DTT) was prepared as a 5X stock. Oligonucleotide annealing buffer (10 mM Tris pH 7.5 @ 25°C, 50 mM KCl, 0.1 mM EDTA) was prepared as a 10X stock. Ligase reaction quench (50 mM EDTA, 0.1% Triton^®^ X-100) was prepared at 1X.

### Preparation of DNA Substrates

HPLC-purified, synthetic single-stranded oligonucleotides were obtained from Integrated DNA Technologies (IDT; Coralville, IA) as lyophilized solids. Oligonucleotides were stored as 100 μM stocks in 1X oligonucleotide annealing buffer using mass specifications provided by IDT. The nicked dsDNA (ds-nDNA) substrate was prepared by combining 1 molar equivalent of the 3’-6-Carboxyfluorescein (FAM)-labeled downstream fragment “p-DNA” with 1.1 molar equivalents of both the upstream unlabeled fragment “DNA-OH” and the complementary splinting strand “splint-DNA” in DNA annealing buffer (S1 Table). This mixture was heated to 95°C and cooled to room temperature slowly over at least 2 hours. The concentration of the annealed ds-nDNA stock is expressed in terms of the FAM-labeled fragment. All ds-nDNA constructs contain both a 3’-OH and 5’-PO4, unless specified otherwise in the figure legend. All other dsDNA substrates were annealed as above in a 1:1 ratio of the two complementary strands.

### Steady State Ligation Assay

Standard ligation assay mixtures were composed of 1X ligase buffer, 5–100 pM ligase, and FAM labeled ds-nDNA concentrations as indicated in each figure legend, with a reaction volume of 200 μL. Reactions were performed at 16°C for T4 DNA ligase, 25°C for PBCV-1, T3 and T7 DNA ligases, and 55°C for *Tth* DNA ligase. Components were gently mixed by pipetting and incubated at reaction temperature for 5 minutes prior to initiation by the addition of the ds-nDNA substrate. Reactions were quenched by a 1:1 vol:vol addition of ligase reaction quench at time points as indicated in each figure legend. The ligated product was analyzed by capillary electrophoresis as described previously.[27–30]

### Determination of k_cat_ and K_m_

The method of initial rates was utilized to determine the Michaelis-Menten parameters k_cat_ and K_m_. Initial rates were determined through linear fits to enzyme reaction profiles in their initial linear phase (30% product conversion or less). Initial rates were determined over a range of FAM-labeled nicked dsDNA substrate concentrations (0.0025–1 μM). The rates were plotted against their respective substrate concentrations and fit with the Michaelis-Menten equation (Eq 1) to extrapolate the kinetic parameters:

$$\frac{V_{0}}{\left[E\right]}=\frac{k_{cat}*\left[S\right]}{K_{m}+\left[S\right]}$$ (1)

where K_m_ is the Michaelis constant, [S] is the substrate concentration, [E] is the enzyme concentration, V_0_ is the initial rate and k_cat_ is the turnover number. In the event of observed inhibition by higher concentrations of substrate, the data was fit by either a substrate inhibition model (Eq 2):

$$\frac{V_{0}}{\left[E\right]}=\frac{k_{cat}*\left[S\right]}{K_{m}+\left[S\right](1+\frac{\left[S\right]}{K_{i}})}$$ (2)

where K_i_ is the substrate inhibition constant, [31] or a competitive substrate inhibition model for an ordered Bi-Bi Ping-Pong reaction:

$$\frac{V_{0}}{\left[E\right]}=\frac{k_{cat}*\left[S\right]}{K_{m}+\left[S\right](1+\frac{K_{mATP}}{\left[ATP\right]}*\left(1+\frac{\left[S\right]}{K_{i}}\right))}$$ (3)

where k_cat_ is the turnover number, K_m_ is the Michaelis constant for substrate binding K_mATP_ is the Michaelis constant for ATP binding, and K_i_ is the inhibitor binding constant [32]. All nonlinear least squares data fitting was performed with the KaleidaGraph software (Synergy Software, Version 4.5.1).

### Ligation Assays in the Presence of Non-template Inhibitors

Inhibition of DNA ligation rates was examined in the presence of large concentrations of various unlabeled, single-stranded DNA (ssDNA) or dsDNA constructs (40mer, 75mer, Puc19). Assays were performed with 20 nM Fluorescein (FAM)-labeled ds-nDNA substrate in the presence of inhibitor DNAs and ligase concentrations (ranging from 5–100 pM) as specified in each figure legend. In order to extrapolate an inhibitory constant, a range of concentrations of a 75 bp long dsDNA rod (I-75-dsDNA) was added to reactions containing 20 nM labeled substrate ds-nDNA and incubated under standard buffer conditions with 25 pM T4 DNA ligase. All reactions were performed a minimum of three times; reported initial rates are the average of the individual experiments, and error is reported as one standard deviation.

The initial rates for the inhibited reactions were normalized to the uninhibited reaction rate [33, 34], and the [Inhibitor]/[Substrate] relationship was fit using a competitive inhibition model (Eq 4):

$$V_{0}=\frac{V_{max}}{\left(\frac{K_{m}}{\left[Substrate\right]}*\frac{K_{m}*\left[Inhibitor\right]}{K_{i}*\left[Substrate\right]}+1\right)}$$ (4)

where V_0_ is the observed rate for the inhibited reaction, V_max_ is the maximal rate for the uninhibited reaction. K_m_ is the Michaelis constant for the substrate and K_i_ is the inhibition constant. A K_d_ for the inhibitor is calculated by multiplying the K_i_ value by the number of nonspecific binding sites on the inhibitor (N), calculated with (Eq 5):

N=2(L−l+1) (5)

where L is the total length of the oligonucleotide, l is the estimated DNA-binding footprint size for the ligase.[20, 21, 35] The value (N) is multiplied by a factor of two to account for the possibility of binding to either the 3’-5’ or 5’-3’ strands. This calculation assumes that the ligase would bind with equal affinity to all non-specific binding sites.

#### Preparation of Deadenylylated T4 DNA Ligase

Deadenylylated T4 DNA ligase was prepared by a 1-hour reaction of the enzyme in deadenylylation buffer (50 mM Tris PH 7.5, 10 mM DTT, 10 mM Mg^2+^, 1 mM sodium pyrophosphate) and subsequent dialysis into a storage buffer (50 mM Tris pH 7.5, 10 mM DTT, 10 mM Mg^2+^) overnight at 4°C. The dialysis buffer was changed a minimum of 3 times, however the initial buffer change was limited to a 1-hour incubation to prevent precipitation of magnesium pyrophosphate; all subsequent buffer changes were performed for a minimum of 4 hours. Adenylylation state of the ligase was confirmed by monitoring the ligation of a 10 nM sample of 3’-FAM labeled ds-nDNA 75mer substrate with 40 nM enzyme in the absence of ATP. We observed 1.54% of the 10 nM DNA ligated after a 30-minute reaction at 16°C, indicating that the preparation was >99.6% deadenylylated.

### Rapid Quench-Flow Assay for Detection of dsDNA Induced Inhibition of Enzyme Self-Adenylylation

Rapid quench experiments were performed using a KinTek Rapid Quench Flow (RQF)-3 (Kintek Corporation, Austin TX) instrument. Samples were prepared with 5 μM deadenylylated T4 DNA ligase with and without 5 μM or 50 μM I-75-dsDNA in 1X ATP-free ligase reaction buffer in syringe A and ATP (2 mM added to 1X ATP-free ligase reaction buffer containing 200 μCi of [α-^32^P] ATP/mL (Perkin Elmer, Waltham, MA)) solution in syringe B. Drive syringes contained 1X ATP-free ligase reaction buffer, and a quench composed of 250 mM EDTA plus 0.25% SDS was used. Collected time points were treated by passing 50 μL through a Centrisep^®^ 10 spin column equilibrated in 50 mM Tris pH 7.5, 0.1% SDS. The flow-through was mixed with 8 μL of 6X Blue Gel Loading Dye (New England Biolabs), and 20-μL aliquots were loaded onto (4–20%) Tris Glycine SDS-PAGE gels and run at 120 V for 30 minutes. The gels were Coomassie stained, the protein band was cut out, and the amount of ^32^P-AMP incorporation was counted on a Tri-carb 2900TR liquid scintillation counter (Perkin Elmer, Waltham, MA). Maximal enzyme adenylylation was determined by the amount of ^32^P detected after a 60-second reaction time point. Reaction progress is reported as the fraction of this maximal adenylylation.

Self-adenylylation single turnover rate was determined under saturating ATP conditions by fitting the data with a single exponential equation (Eq 6):

$$Y=Ae^{-kt}$$ (6)

where A is the reaction amplitude, and k is the observed single turnover rate. Reported experimental data are the average of a minimum of three replicates, and error reported is the standard error of the measurements.

### Electrophoretic Mobility Shift Assay

The downstream fragment of the 75mer ds-nDNA substrate utilized in the EMSA experiments (p-DNA-noFAM/PO_4_) was 5’ PO_4_ labeled using T4 PNK (New England Biolabs) with [γ^32^P] ATP (3000 Ci/mmol) (Perkin Elmer, Waltham, MA) following standard manufacturer’s protocols. The substrate was assembled as indicated above. EMSAs were performed in 20 μL total volumes with the addition of increasing concentrations of deadenylylated T4 DNA ligase (as indicated) into the 5’ ^32^P-labeled 75mer-ds-nDNA substrate, or stepwise addition of a non-labeled, non-nicked 75 dsDNA rod (I-75-dsDNA) into a prebound T4 DNA ligase, 5’ 32P-labeled 75mer-ds-nDNA complex. Reactions were performed in ligase-binding buffer (50 mM Tris pH 7.5, 5 mM DTT, 5 mM Mg^2+^, 1 mM sodium pyrophosphate, 5% Glycerol). Prior work reported that adenylylated ligase, when in large excess, was still able to slowly seal a nicked substrate in reaction conditions lacking added Mg^2+^, even in the presence of 5 mM EDTA.[27] Due to the high concentrations of enzyme required to form the complex, sodium pyrophosphate was included in the binding buffer to eliminate any ligase-adenylate and ensure no portion of the substrate would be ligated during the EMSA. Sodium pyrophosphate has been previously reported not to influence formation ligase DNA complex by EMSA.[10] Binding reactions were incubated at room temperature for 20 minutes prior to being loaded on 6% native Novex^®^ TBE acrylamide gels (Life Technologies, Carlsbad CA) and electrophoresed for 30 minutes at 180V. The gels were imaged using a Typhoon 9400 imager (GE Life Sciences, Pittsburgh PA) and analyzed with the included Image Quant TL software (V 7.0).

## Results

### T4 DNA Ligase Steady State Ligation Rate is Inhibited by Increased Substrate Concentration

**Fig 2:**
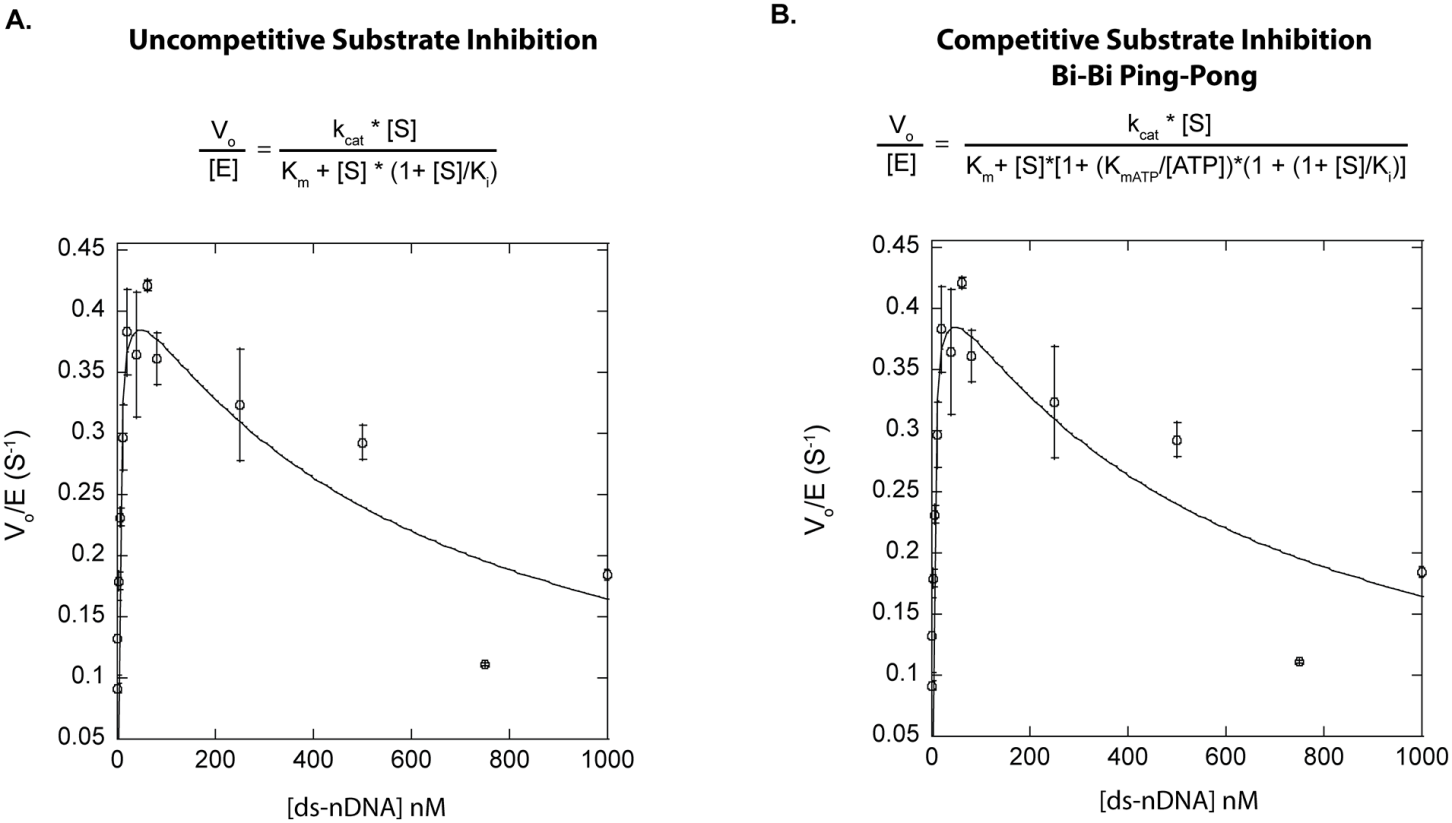
k_cat_/K_m_ Curve for T4 DNA Ligase. The data was obtained through titration of increasing concentrations of a 75mer-ds-nDNA substrate, reacted at 16°C to determine initial reaction rates. T4 DNA ligase concentrations used were 25 pM– 100 pM. The initial rates were plotted against their respective substrate concentrations and fit by: **A**. a classical uncompetitive substrate inhibition model (Eq 2), where k_cat_ and K_m_ Values of 0.44 s^-1^ ± 0.3 s^-1^ and 4 nM ± 1 nM respectively, were determined. The K_i_ value for substrate inhibition was calculated to be 590 nM ± 170 nM. **B**. A competitive substrate inhibition for a Bi-Bi Ping-Pong mechanism (Eq 3) k_cat_ and K_m_ values of 0.48 s^-1^ ± 0.3 s^-1^ and 4 nM ± 1 nM respectively, were determined. The K_i_ value for substrate inhibition was calculated to be 54 nM ± 15 nM. All data points are the average of at least three independent experiments, and the error reported is the standard deviation for the replicates.

Initial studies to determine the kinetic parameters for nick sealing by a variety of ligases (T4, PBCV-1 and *Tth*) showed a significant reduction in initial velocity at high concentrations of ds-nDNA substrate, indicative of a previously unobserved substrate inhibition effect in these enzymes. (Fig 2A, S1 Fig). The data were fit to a classic uncompetitive substrate inhibition model (Eq 2) to allow estimation of the Michaelis constant (K_m_) for productive substrate binding and the K_i_ for the inhibitory substrate interaction. The K_m_ values were determined to be 4 nM ± 1 nM for T4 (Fig 2A) 1.6 nM ± 0.9 nM for *Tth*, and 2.2 nM ± 1.7 nM (S1 Fig) for PBCV-1 respectively. The K_m_ values for T4 and PBCV-1 DNA ligases were in line with previously determined values.[27, 28] The K_m_ for *Tth* ligase was lower than previously reported (87 nM), but is likely due to use of a lower reaction monovalent cation concentration (25 mM K^+^ in *Taq* DNA ligase buffer versus 100 mM K^+^ in the Barany study).[36] In all cases, the inhibition equilibrium constant (K_i_) of the 75mer-ds-nDNA substrate was estimated to be 200–500 nM for these ligases. In addition to uncompetitive substrate inhibition, the inhibition could also potentially be explained by a competitive model, with nicked substrate binding to deadenylylated ligase inhibiting the self-adenylylation reaction through blocking binding of ATP [6, 37]. Thus, the T4 substrate inhibition data was also fit with a competitive substrate inhibition model for an ordered Bi-Bi Ping-Pong mechanism (Eq 3). This model can fit the data with a similar quality of fit (R^2^ = 0.89 vs. R^2^ = 0.89 for uncompetitive inhibitions). Using the previously reported K_mATP_ of 100 μM, it was determined that the K_m_ for substrate binding is 4 nM ± 1 nM, while the K_i_ is 54 ± 15 nM. [38] Thus, this model predicts the same K_m_ but requires tighter inhibitory binding. While the best fits to the data sets were obtained applying a substrate inhibition model (R^2^ = 0.89), deviations from an ideal fit and similar fit quality obtained by a competitive model suggested the mechanism of inhibition was likely more complex than can be accounted for with a simple substrate inhibition model.

### T4 DNA Ligase is Inhibited by Mass Quantity Rather Than Structure of DNA

**Fig 3:**
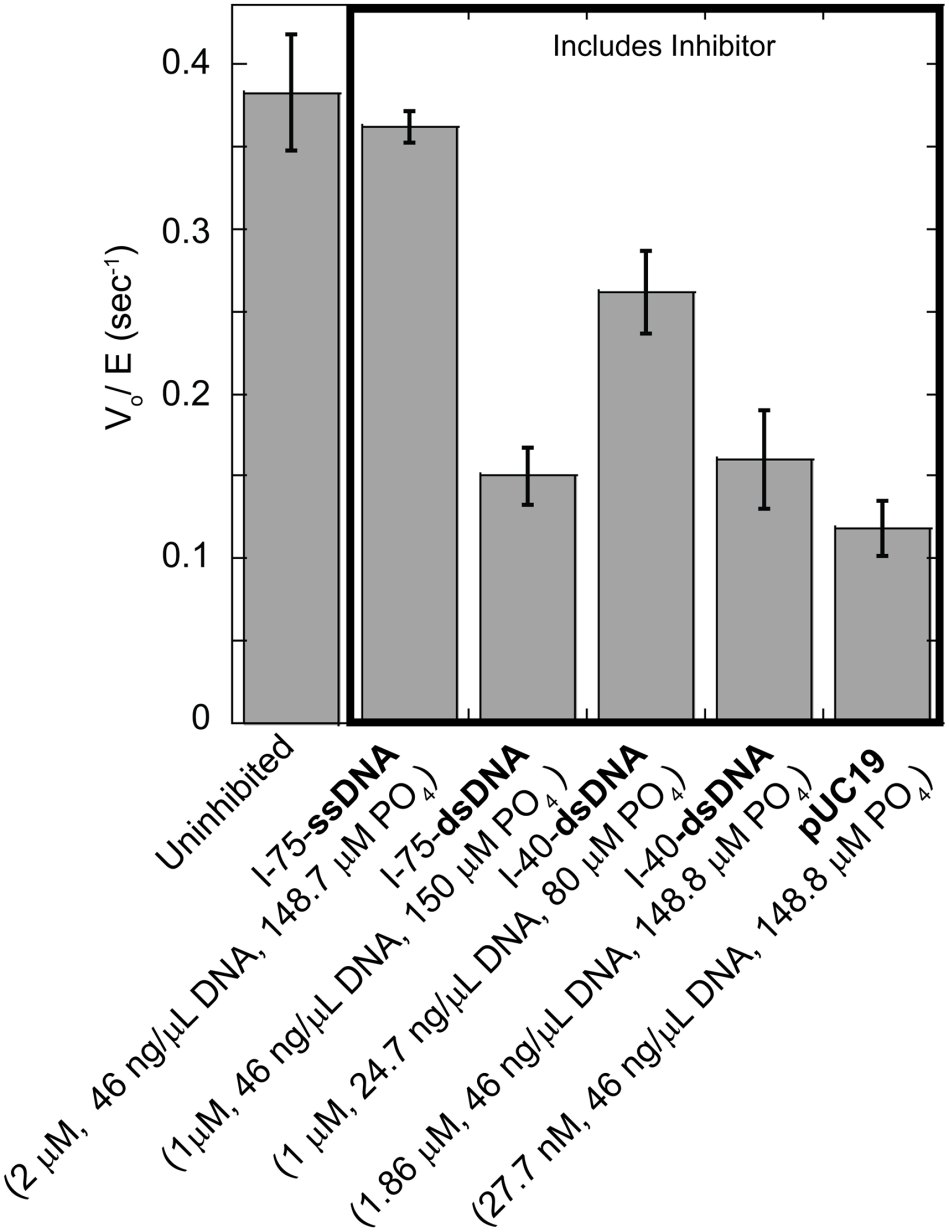
Various Inhibitors Effects on Rate of Nick Sealing. Various concentrations of dsDNA substrates were utilized as potential inhibitors of the T4 DNA ligase steady state ligation reaction on 20 nM of the 75mer-ds-nDNA substrate. All reactions were performed in the presence of 25 pM of T4 DNA ligase, a minimum of three times at 16°C. Error reported is the standard deviation for the replicates.

To determine whether the observed substrate inhibition of T4 DNA ligase was due to the nick concentration or to the non-substrate dsDNA portions of the substrate, ligation reactions were performed with a low 75mer-ds-nDNA FAM-labeled substrate concentration (20 nM) and large excess (1–2 μM) of unlabeled DNA substrates with different structure (linear ssDNA, linear dsDNA of two lengths, and circular dsDNA) to act as potential inhibitors. The presence of non-nicked, double-stranded 75mer (I-75-dsDNA) linear oligo, with a sequence unrelated to the nicked substrate, was observed to cause a decrease of initial velocity of steady state nick sealing per unit enzyme V_0_/E from 0.38 s^-1^ ± 0.04 s^-1^ to 0.15 s^-1^ ± 0.02 s^-1^. When an equivalent 1 μM concentration of a shorter 40mer dsDNA oligo (I-40-dsDNA) was included as the inhibitor, the V_0_/E was decreased to 0.26 s^-1^ ± 0.02 s^-1^, a less inhibitory effect than the same molar concentration of the I-75-dsDNA. Here, the total amount of DNA present for the I-75-dsDNA is greater than that of the I-40-dsDNA, as is illustrated by comparing the phosphate concentration for the two reactions (150 μM for the 1 μM I-75-dsDNA vs. 80 μM for the 1 μM I-40-dsDNA). Interestingly, when an equivalent concentration by mass per volume (46 ng/μl) of the I-40-dsDNA was tested, such that both I-75-dsDNA and I-40-dsDNA reactions included equivalent DNA phosphate concentrations, a more similar V_0_/E of 0.16 s^-1^ ± 0.03 s^-1^ was observed (Fig 3). Additionally an equivalent concentration by mass per volume (46 ng/μl) of the circular dsDNA pUC19 plasmid was also used as an inhibitor, and yielded a similarly inhibited V_0_/E value of 0.11 s^-1^ ± 0.02 s^-1^ (Fig 3). Taken together, these results suggested that the inhibition is proportional to the total amount of dsDNA in the reaction, and was not dependent on end binding. A similar inhibitory effect was not observed for an equal molar amount of I-75-ssDNA (Fig 3), indicating the effect was specific to dsDNA. A similar pattern of inhibition on ds-nDNA sealing rates was observed for several other DNA ligases, though the strength of the inhibition varied from stronger than T4 (PBCV-1) to essentially uninhibited at the concentrations tested (*Tth*) (S2 Fig). As a control, two non-ligatable nicks, which would be expected to bind competitively in place of ligatable substrate, were tested for their inhibitory effect (S3 Fig). Indeed, the ligation rate was nearly completely inhibited by 1 μM of an unlabeled I-75-dsDNA containing a nick lacking either a 5’PO_4_ or 3’OH. Thus T4 DNA ligase appears to be inhibited by dsDNA independent of the concentration of ends, but the interaction with the dsDNA backbone is clearly weaker than binding to a non-ligatable nick substrate analogue.

### Non-Nicked dsDNA is Able to Compete for T4 ds-nDNA-Binding

**Fig 4:**
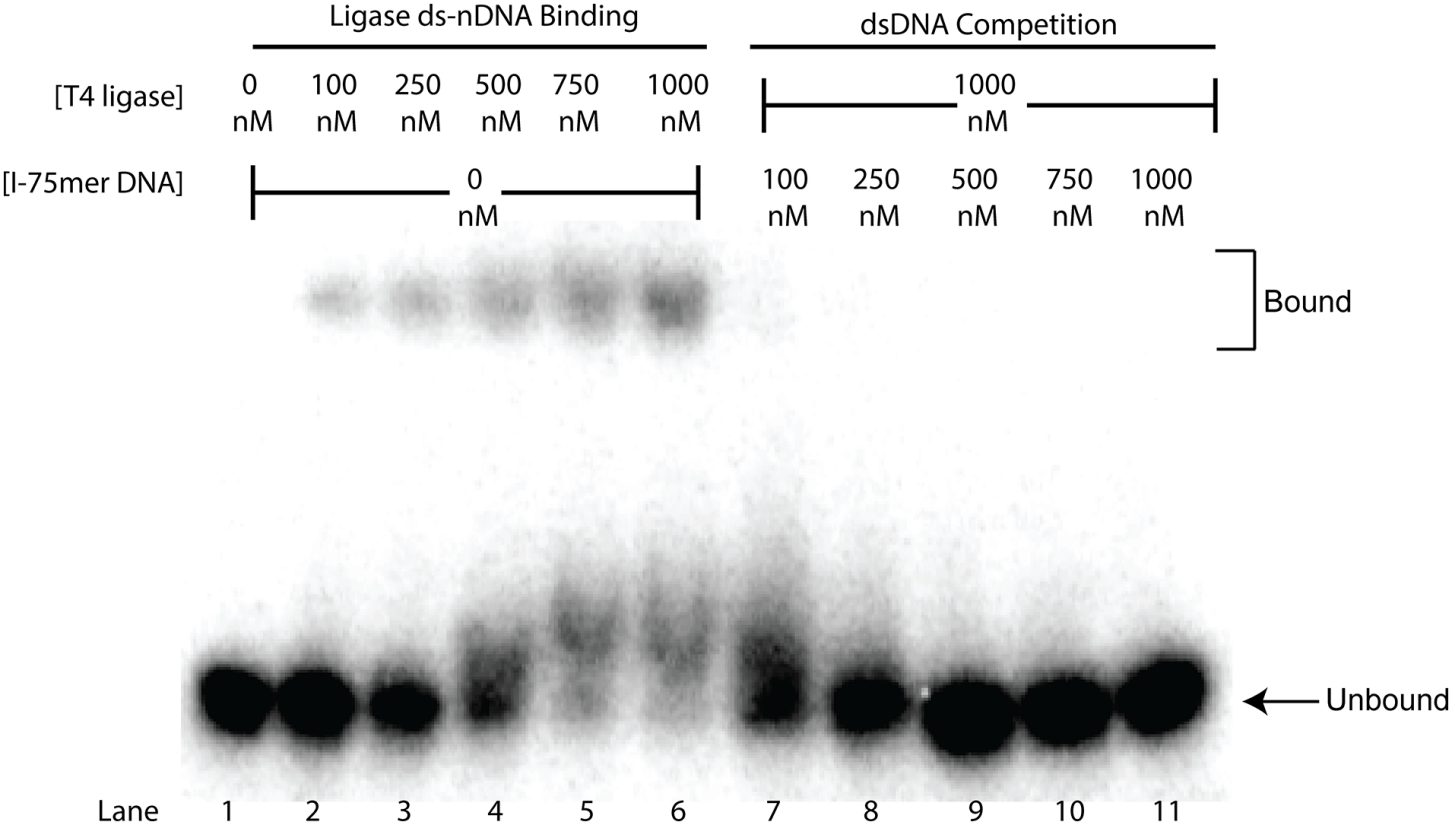
Competition for ds-nDNA-Binding by dsDNA. Lane one contains 4 nM of the 75mer-ds-nDNA substrate alone, lanes 2–6 show shifting of the 4 nM substrate into a completely bound state as the concentration of T4 DNA ligase is increased from 100 nM– 1000 nM. Lanes 7–11 are of a titration of increasing concentrations of the unlabeled I-75-dsDNA oligo into a reaction containing 4 nM labeled nicked substrate and 1000 nM T4 DNA ligase. EMSA reactions were all performed and electrophoresed at room temperature (22°C).

Electrophoretic mobility shift assays (EMSAs) were performed to examine the effect of increased non-substrate dsDNA concentrations on the ability of the ligase to bind its 75mer-ds-nDNA substrate. ATP-free reaction conditions and deadenylylated T4 DNA ligase were used to prevent ligation of the nicked substrates upon binding to the ligase. The substrate was first shown to be effectively bound by the ligase (Fig 4, **lanes 2–6**). The highest concentration of T4 DNA ligase used (1 μM) was then incubated in the presence of the labeled ds-nDNA and increasing concentrations of non-labeled, non-substrate I-75-dsDNA. Even the lowest concentration of dsDNA used (100 nM) was able to effectively compete the ligase from its preferred ds-nDNA substrate, with higher concentrations effectively completely competing the ligase off the nicked substrate. (Fig 4, **lanes 7–11**). This result is consistent with competitive binding by the ligase to non-substrate DNA, a likely mechanism of the observed inhibition of nick ligation rates.

### Quantitative Determination of the K_i_ of dsDNA Through Competitive Inhibition Experiments

**Fig 5:**
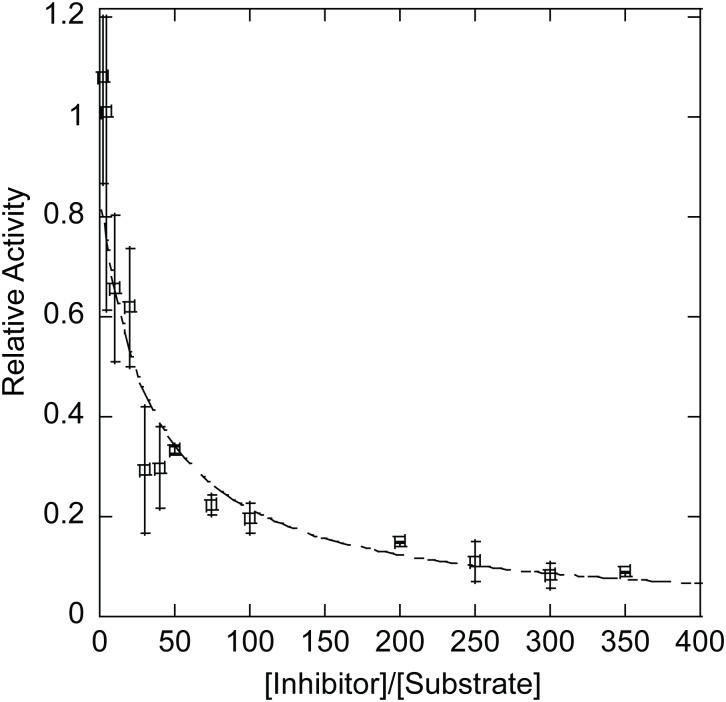
Effect of Increasing DNA Concentration on ds-nDNA Sealing Rate. Competitive inhibition fitting utilizing Eq 4. The K_i_ for the addition of a I-75-dsDNA substrate was determined to be 100 nM ± 20 nM. The affinity per base pair can also be calculated utilizing Eq 5. Utilizing a binding footprint size of 24 bp, the K_i_ is calculated as 10 μM ± 2 μM. All reactions were performed a minimum of three times at 16°C. Error reported is the standard deviation for the replicates.

To test the competitive inhibition theory, a constant concentration of 75-n-dsDNA nicked substrate was reacted with a range of concentrations of non-substrate I-75-dsDNA and the effect on initial velocity measured. In order to extract the inhibition constant of dsDNA on T4 DNA ligase, a competitive inhibitor fit was utilized, (Eq 4) where the observed rate with inhibitor was normalized to the reaction rate without inhibitor (Fig 5). From this fit, along with the previously determined K_m_ value calculated for T4 DNA ligase of 4 nM ± 1 nM, K_i_ for the system of 100 nM ± 20 nM for the I-75-dsDNA inhibitor was extracted, indicating stronger inhibition by non-substrate DNA than the ~200–500 nM K_i_ determined for inhibition by the substrate. The K_d_ for the minimal bound portion of double-stranded DNA can be calculated by utilizing Eq 5 and multiplying the K_i_ by the number of binding sites (N) on the I-75-dsDNA, which was equal to ~100, using an estimated binding footprint size of 24 bp, resulting in a K_d_ of 10 μM ± 2 μM per binding site. This analysis was also performed utilizing the shorter I-40-dsDNA where a K_i_ of 200 ± 30 nM was determined (S4 Fig). The K_d_ for the minimal bound portion of the I-40mer-dsDNA was calculated using the number of binding sites for T4 DNA ligase on a 40 bp linear oligonucleotide (N = 34), and was determined to be 7 ± 1 μM, consistent with the value determined for the longer substrate. The estimated binding footprint size used was based upon the binding size observed for other crystalized DNA ligases, as there is no available structure for T4 DNA ligase.[20, 21, 35] The accuracy of this determined K_d_ was reliant on determination of an accurate K_m_ for the interaction between the ligase and nicked substrate. The K_m_ which was utilized for this calculation was derived from the substrate inhibition fit for Fig 2A. The data fitwell to this competitive inhibition model, suggesting that non-substrate DNA inhibits largely through competitive binding with nicked substrate.

### Non-nicked dsDNA Inhibits Enzyme Self-Adenylylation in the Absence of Nicked Substrate DNA

**Fig 6:**
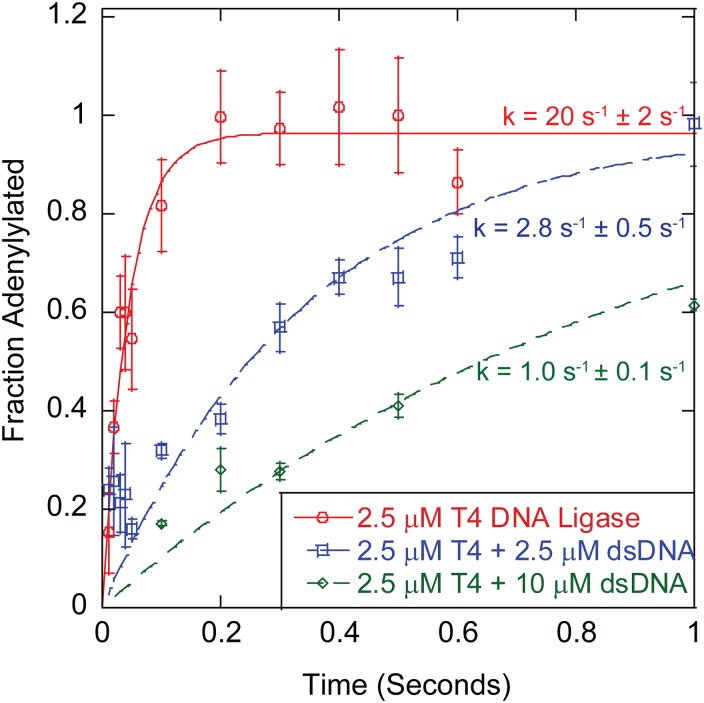
Effect of Inhibiting dsDNA on Enzyme Self-Adenylylation Rate. The determined rates for self-adenylylation of an uninhibited reaction, 2.5 μM T4 DNA ligase (red) and 2.5 μM T4 DNA ligase and inhibited reactions 2.5 μM DNA (blue) and 10 μM DNA (green). The reactions were fit to a single exponential equation (Eq 6) to determine the reaction rate. The uninhibited reaction was determined to have a single turnover rate of 20 s^-1^ ± 2 s^-1^. While the 2.5 μM inhibited reaction had a single turnover rate of 2.8 s^-1^ ± 0.5 s^-1^and the 10 μM inhibited reaction had a single turnover rate of 1.0 s^-1^ ± 1 s^-1^. All reactions were performed a minimum of three times at 16°C. Error reported is the standard error for the replicates.

Inhibition of ligation could result solely from non-nicked dsDNA blocking binding of nicked substrate; however, ligation rate could also be influenced by blocking the ability of the ligase to bind or react ATP in the self-adenylylation reaction. A single turnover assay was utilized to examine the rate of T4 DNA ligase self-adenylylation in the presence or absence of non-nicked, non-substrate I-75-dsDNA. Deadenylylated ligase was reacted with α^32^P-ATP, detecting the conversion of the ligase to the adenylylated form through the incorporation of radioactivity (Fig 6). In the absence of DNA, a self-adenylylation reaction rate of 20 s^-1^ ± 3 s^-1^ was observed, comparable to previously published data.[3, 27] Upon inclusion of 2.5 μM I-75-dsDNA, a 7-fold decrease in self-adenylylation rate to 2.8 s^-1^ ± 0.5 s^-1^ was observed (Fig 6), suggesting that dsDNA is able to interact with the deadenylylated form of the enzyme in an inhibitory mode. Increasing the dsDNA concentration in the reaction to 10 μM resulted in further reduction of the self-adenylylation rate to 1.0 s^-1^ ± 0.1 s^-1^ (Fig 6). Thus, non-substrate dsDNA can inhibit single-turnover self-adenylylation as well as the turnover rate of nick ligation.

## Discussion

**Fig 7:**
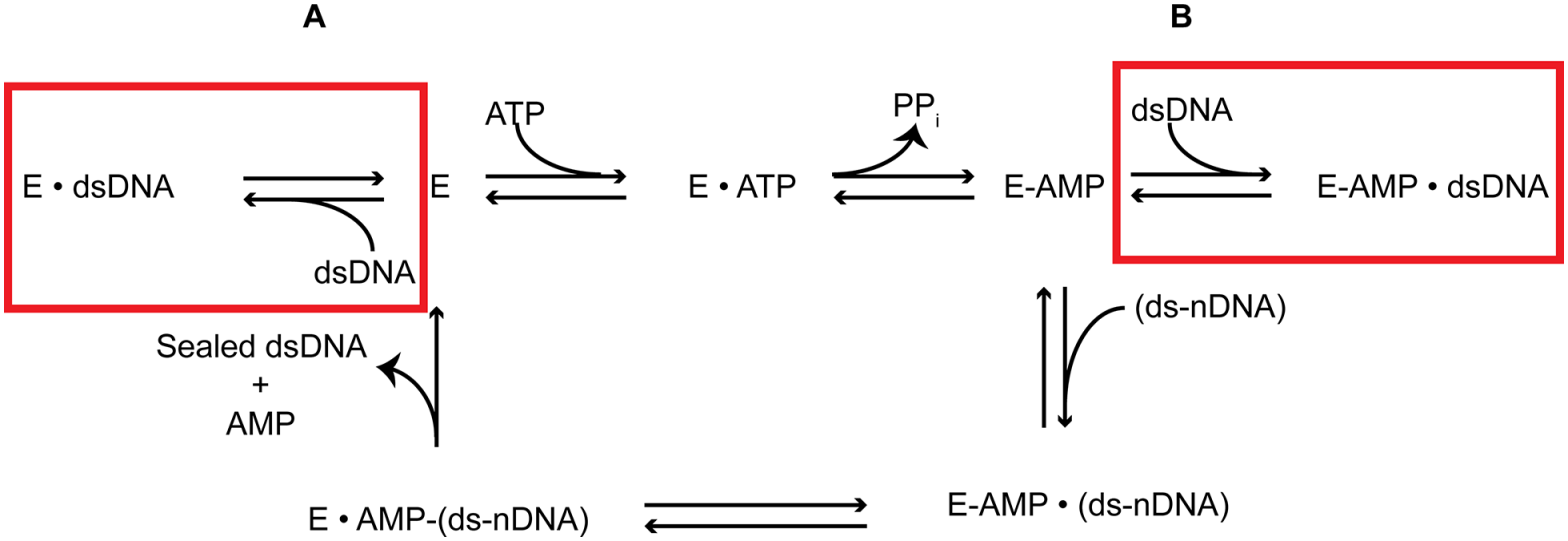
T4 DNA Ligase Reaction Model. Modified reaction pathway to include the newly observed reactions in the previously described DNA ligation pathway that are inhibited by the presence of non-nicked dsDNA. **A**. Non-nicked dsDNA can bind to the deadenylylated form of the enzyme inhibition formation of the adenylylated form of the enzyme. **B**. Non-nicked dsDNA binds to the Lig-AMP form, preventing complexation with its preferred ds-nDNA substrate.

This study describes two significant forms of inhibition for T4 DNA ligase: inhibition by non-nicked dsDNA and inhibition by nicked substrate DNA. We have created a modified reaction pathway illustrating the steps in the ligation reaction pathway inhibited by non-nicked dsDNA as reported in this work (Fig 7). Here we highlight that non-nicked dsDNA is able to inhibit both the enzyme self-adenylylation step, either by competing for ATP binding or acting as an uncompetitive inhibitor (Fig 7A), as well as serving as a competitive inhibitor to the binding of the ds-nDNA substrate (Fig 7B). The concentrations of dsDNA shown to be highly inhibitory (46 ng/μL) in this work have relevance to both cellular functioning of these enzymes as well as in molecular biology protocols. The average cellular DNA concentration of an actively replicating *E*. *coli* cell is 1.83*10^4^ ng/μL.[39] While this value does not take into consideration the amount of cellular dsDNA made inaccessible through involvement in complexes with replication machinery, it is clear that cellular dsDNA concentrations would be high enough such that inhibition would play a role in cellular DNA ligase functioning. DsDNA inhibition likely also has an effect in commonly used molecular biology protocols, for example, the maximal recommended DNA concentration utilized in the ligation step for Next Generation Sequencing library preparation is ~20 ng/μL (NEB Ultra II), while the recommended DNA concentration in a standard sticky-end ligation is 4.38 ng/μL (NEB T4 DNA ligase ligation protocol). While these concentrations are lower than the inhibitory dsDNA concentration utilized in this work, they are still sufficiently high enough to be impactful to ligase function. This observed inhibition may also play a role in the increased difficulty observed for the ligation of sticky-ended fragments into large plasmids, where the lower concentrations of reactive ends and increased length of dsDNA would inhibit the functioning of T4 DNA ligase, commonly used in these protocols.

Prior characterization of DNA ligase binding to DNA through EMSAs indicated that both a 5’-phosphorylated nicked substrate and the adenylylated form of a DNA ligase were required for the formation of a stable complex.[10, 18, 35, 40] However, these early findings are at odds with the proposed mechanisms for DNA ligase nick sensing, which implies the need for interaction between the ligase and non-nicked DNA. In the mechanistic depiction by Rossi *et al*., the authors proposed a three-step nick-sealing model, where the first step was nick-scanning via a transient association of the adenylylated form of the ligase with duplex DNA. A later model based on the PBCV-1 ligase crystal structure postulated that nick recognition involves the bending of the dsDNA at the break by the ligase, accompanied by a transition of standard B-form DNA to A-form for the two nucleotides on either side of the nick.[10, 21, 23] A nick site possesses the necessary flexibility allowing for the bending transition and stable formation of the ligase-AMP-DNA complex, while dsDNA will not deform as readily.[22] Both of these models would necessitate a transient interaction with dsDNA by the ligase in order to allow for the interrogation of the DNA for breaks in the phosphodiester backbone.

Previous characterization of T4, *Tth*, and PBCV-1 DNA ligases, did not show these two inhibition effects. [27, 36, 41, 42] However, in these studies the substrate concentrations tested to measure the Michaelis-Menten parameters (T4: max 50 nM substrate, *Tth*: 20 nM substrate, PBCV-1: 100 nM substrate) were likely too low to clearly show the inhibition effect, while the broader substrate concentration range used in this study made the substrate inhibition effect much more apparent. [27, 36, 41, 42] While observed here in multiple enzymes, substrate inhibition may not be a general feature of all DNA ligases. For example, it has been reported that human DNA ligase I and human DNA ligase III remain uninhibited even in the presence of 4 μM nicked substrate.[7]

Recent reports on the activity of *H*. *influenza* and *S*. *pneumoniae* DNA ligases also described a significant substrate inhibition effect.[6, 37] For these enzymes, it was hypothesized that the underlying mechanistic reason for the inhibition was the competitive binding of deadenylylated enzyme to the nicked substrate, thus inhibiting enzyme self-adenylylation.[6, 37] In this study, EMSAs performed with a deadenylylated form of T4 DNA ligase clearly demonstrated that stable binding of nicked DNA does occur for this ligase (Fig 4), providing direct evidence for potential inhibition of enzyme self-adenylylation through this mechanism. An important corollary to this substrate inhibition mechanistic hypothesis is that the studies of *H*. *influenzae* and *S*. *pneumoniae* DNA ligases were observed under reaction conditions where the cofactor (ATP or NAD^+^) concentrations were substantially lower than would be found in the cell, resulting in a large pool of deadenylylated ligase available to bind the nicked substrate. Thus, a large potential effect on ligase self-adenylylation rates might be expected.[6, 37] By contrast, the T4 phage’s *E*. *coli* host has a free intracellular ATP concentration of 1.5 mM ± 1.2 mM, [43] comparable to the 1 mM ATP concentration used in this study. The ATP concentration utilized here results in a very high degree of enzyme adenylylation, similar to or greater than what would be observed *in vivo*.[44] Additionally, fast timescale measurements have shown that the binding of ATP is expected to be very fast at 1 mM ATP and effectively irreversible, implying a true KD much tighter than the 100 μM K_m_ reported previously.[38, 44] Thus, while the steady state data can be fit well by both a simple substrate inhibition model as well as a competitive substrate inhibition model for a Bi-Bi Ping-Pong reaction mechanism, using tighter ATP binding values implied by the fast timescale studies in the competitive substrate inhibition fit results in unrealistically tight K_i_ values for DNA substrate inhibitor binding. This analysis fact suggests that, for T4 DNA ligase at least, inhibition of enzyme adenylylation is unlikely to be the major source of substrate inhibition, and additional uncompetitive inhibitory modes may be at work. For example, binding of a second substrate molecule to the adenylated enzyme-substrate complex could inhibit reaction rates, or binding of substrate to an enzyme-product complex could inhibit release of one or both. As such, we believe that the uncompetitive fit more accurately depicts the effect of high nDNA substrate concentrations have on T4 DNA ligase.

In addition to substrate inhibition, it was observed that T4 DNA ligase was inhibited by increased concentrations of either linear or circular dsDNA lacking nicks (Fig 3). Inhibition by non-substrate, non-nicked dsDNA was also observed here for other DNA ligases (S2 Fig, PBCV-1, T3, T7); however, the degree to which these ligases were inhibited is variable. Additionally, for *Tth* DNA ligase, little or no inhibition was observed for either linear or circular dsDNA (S2 Fig), nor was inhibition found in previous studies on *Thermococcus kodakarensis* DNA ligase. [45]. Assuming random binding with a footprint of 24 bp for T4 DNA ligase, the K_d_ was ~8–10 μM per putative non-nicked dsDNA-binding site (Fig 5 **and** S4 Fig). It is likely that this interaction is transient, as we were unable to observe a stable complex of T4 DNA ligase and non-substrate dsDNA by EMSA, similar to what had been observed in previous DNA-binding experiments.[17, 18] However, increasing concentrations of dsDNA were observed to compete with a nick-containing substrate for binding to the enzyme (Fig 4), illustrating that dsDNA can serve as a competitive inhibitor for substrate ds-nDNA-binding. As the structure of the dsDNA, short blunt fragments of different lengths or circular pUC19 DNA, did not seem to affect the degree of inhibition for ligation by T4 DNA ligase, the mechanism of inhibition is most likely competitive, transient binding randomly along the DNA backbone.

Recent work on Human DNA ligase III showed that this enzyme was able to form stable bridging complexes between two blunt-ended dsDNA fragments, displaying strong binding in the absence of nicked sites or phosphates.[46] As T4 DNA ligase is also known to possess effective blunt DNA fragment ligation properties, it is possible that the inhibition from non-nicked dsDNA could be due to similar end-specific binding. For ligases that are more weakly inhibited by dsDNA (T3, T7) a stronger inhibition was in fact observed from linear dsDNA than from circular plasmid DNA. However, for the ligases more strongly inhibited by dsDNA (T4 and PBCV-1), a strong preference for end binding does not seem likely, as equal concentrations of backbone phosphates were equally inhibitory regardless of structure (I-75-dsDNA oligo, I-40-dsDNA oligo, or pUC19 circular DNA) (Fig 3, S2 Fig). In these cases, nonspecific backbone binding seems to dominate the inhibitory effect, but these differences suggest that ligases can have different affinity for binding at the ends versus along the backbone, and that the specific affinity for various binding modes may play into details of DNA and nick recognition mechanisms amongst ligases.

In addition to inhibition of steady-state, nick-sealing rates, inhibition of T4 ligase self-adenylylation rate was also observed in the presence of high concentrations of I-75-dsDNA (Fig 6). It is reasonable to hypothesize that this inhibition is either competitive, with dsDNA competing with ATP binding or uncompetitive, with dsDNA-binding to the ligase·ATP complex, slowing the self-adenylylation reaction. Both of these mechanisms are plausible based upon crystal structures of other DNA ligases in complex with their nicked substrates, as bound DNA could trap the ligase·ATP complex in a non-productive state or prevent ATP access, if the closed conformation of the enzyme is induced upon dsDNA-binding.[21, 35, 47] However, more expansive transient state kinetic experiments than those presented in Fig 6 are needed to determine the precise mechanism of this inhibition, as observation based only on the single turnover product formation does not give enough information to distinguish between models.

Despite the >15-fold reduction in T4 DNA ligase self-adenylylation observed in the presence of a large excess (10 μM) of I-75-dsDNA (Fig 6), the rate of self-adenylylation was still too fast compared to nick-sealing rates under similar inhibited conditions to be rate limiting. With an uninhibited steady-state, nick sealing rate of 0.4 s^-1^ ± 0.1 s^-1^ and a significantly slower rate when in the presence of 1 μM dsDNA (0.15 s^-1^ ± 0.02 s^-1^), the rate of nick sealing was still much slower than the inhibited adenylylation rate (1.1 s^-1^ ± 0.1 s^-1^) in the presence of high (10 μM) dsDNA (Fig 6). [27] Thus, while self-adenylylation is inhibited by non-substrate DNA, the binding competition between excess non-substrate DNA and nicked DNA is the likely mechanism to account for the overall inhibition by non-substrate DNA during steady state ligase turnover, particularly at the high ATP concentrations used in this experiment. The inhibition by random binding of non-substrate DNA further suggests a second mechanism for substrate inhibition. As the substrate DNA used in this study contains regions of intact dsDNA, it is reasonable that some inhibition of a theoretical V_max_ could result from ligase-dsDNA-binding events remote from the nick. Ligase-non-nicked DNA-binding was shown to be inhibitory by the non-susbtrate DNA experiments, and could explain reduced turnover rates at high substrate concentation. Interestingly, the effect of off-nick binding in the substrate must be weaker than inhibition from similar concentrations of non-nicked dsDNA, as the turnover rate at 1 μM nick (0.184 s^-1^± 0.004 s^-1^) is faster than the rate for 20 nM nick in the presence of 1 μM I-75 DNA (0.150 s^-1^ ± 0.017 s^-1^). However, some fraction of off-nick binding by the ligase maybe able to resolve to the catalytically relevant complex through hopping or sliding of the enzyme. Thus, only a subset of off-nick binding events by adenylylated ligase would contrtibute the observed substrate turnover rates.

The binding of non-substrate DNA by T4 DNA ligase may result from the enzyme forming a transient, DNA-encircling bound form similar to the nick-bound complexes observed in the crystal structures of PBCV-1, E. coli, and human DNA ligases [20, 21, 35]. The crystal structure of PBCV-1 DNA ligase bound to a sealed nick substrate shows few interactions between the protein site and the product dsDNA, suggesting this binding mode would indeed be a weakly bound complex, consistent with the ~8–10 μM K_d_ observed for T4 DNA ligase interacting with non-substrate DNA.[21] It is also possible that T4 DNA ligase binds non-nicked DNA in a mode distinct from susbtrate binding. T4 DNA ligase possesses multiple domains (OB, DBD, NT) capable of interacting with a DNA substrate, theoretically independently, when the enzyme is in the open conformation, that could, nonetheless, block productive nick binding or conversion to a closed-form, DNA-encircled, nick-binding complex. We hypothesize that the inhibition effect is evidence of a transient initial DNA-binding interaction by which T4 ligase scans DNA for nick sites.[10] In this T-complex/S-complex model previously proposed by Rossi *et al*., as in analogous mechanisms identified in other DNA-binding enyzmes, initial weak binding to random sites on dsDNA, followed by two dimensional scanning via hopping or sliding allow the ligase to search large amounts of DNA in order to rapidly locate its preferred nicked substrate. [34, 48, 49] Ligases binding and scanning remote from nicks or on non-nicked DNA would result in reduced rates of turnover proportional to the amount of ligase bound to DNA in these non-catalytic conformations.

We can conclude that the inhibition of T4 DNA ligase by DNA is likely complex, with contributions from multiple inhibitory mechanisms. Inhibition by substrate must occur at least partially by one or more uncompetitive mechanisms, while inhibition by dsDNA occurs through competition for binding of nicked substrate. As nicked dsDNA substrates contain long stretches of dsDNA it is further likely that the substrate inhibition includes both competitive and uncompetitive components. It is also important to consider that the inhibition of T4 DNA ligase by substrate and non-substrate DNA, even when in the presence of cellularly relevant ATP concentrations is indicative that, given high enough DNA concentrations, inhibition could be impactful in cellular ligase function as well as for *in vitro* systems. It is unclear how ligases in the cell are able to function so effectively when in the presence of high cellular dsDNA concentrations, especially considering the need of ligase turnover during Okazaki-fragment processing. Ligases likely overcome these inhibitory binding events through forming complexes with other proteins involved in the DNA replication process such as replication processivity factor (PCNA/βclamp) to direct them more efficiently to nicked sites.[50–52] Regardless, the binding to non-nicked dsDNA clearly has a significant impact on catalytic functioning of these enzymes *in vitro*. In order to understand the complete mechanism of ligation, we must also fully understand the transient interactions with dsDNA and if and how they can resolve to catalytically active ligase-DNA complexes. Our next area of focus will be the use of fast timescale fluorescence experiments to better elucidate the mechanism behind interactions between the ligase and nonspecific dsDNA to allow for determination of exactly how these interactions influence the rates of the individual steps in the kinetic pathway of DNA ligation.

## Supporting Information

S1 Table DNA Substrates. The above table is a listing of all single- and double-stranded DNA substrates and their respective abbreviations used throughout the manuscript.
(DOCX)

S1 Fig k_cat_/K_m_ Curve for *Tth* and PBCV-1 Ligases. The data was obtained through titration of increasing concentrations of a 75mer-ds-nDNA substrate, reacted at 25°C (PBCV-1), and 55°C (*Tth*) to determine initial rates. Enzyme concentrations used were 25 pM– 100 pM for both PBCV-1 and *Tth* DNA ligase. The initial rates were plotted against their respective substrate concentrations and fit by a substrate inhibition model (Eq 2), where K_m_ values of 1 nM ± 1 nM (PBCV-1), and 2.1 nM ± 0.9 nM (*Tth*) were determined. K_i_ values for the inhibition of each ligase were also determined 115 nM ± 60 nM (PBCV-1), and 200 nM ± 100 nM (*Tth*). All reactions were performed a minimum of three times. Error reported is the standard deviation for the replicates.
(TIF)

S2 Fig Nicked Inhibitor Effects on Rate of Nick Sealing for other DNA Ligases. Various concentrations of dsDNA substrates were utilized as potential inhibitors of the steady state ligation reaction on 20 nM of the 75mer-ds-nDNA substrate. **A**. Reactions with 25 pM of PBCV-1 DNA ligase at 22°C. **B**. Reactions with 50 pM of T3 DNA ligase in T4 DNA ligase buffer at 25°C. **C**. Reaction with 50 pM of T7 DNA ligase in T4 DNA ligase buffer at 25°C. **D**. Reactions with 25 pM of *Tth* DNA ligase in *Tth* DNA ligase buffer at 55°C. All reactions were performed a minimum of three times. Error reported is the standard deviation for the replicates.
(TIF)

S3 Fig Nicked Inhibitor Effects on Rate of Nick Sealing. Effect of unlabeled dehydroxylated (ddC) and dephosphorylated (NoPO_4_) ds-nDNA substrates on the T4 DNA ligase steady state ligation reaction on 20 nM of the 75mer-ds-nDNA substrate. All reactions were performed in the presence of 25 pM of T4 DNA ligase at 16°C. All reactions were performed a minimum of three times. Error reported is the standard deviation for the replicates.
(TIF)

S4 Fig Effect of Increasing I-40-dsDNA Concentration on ds-nDNA Sealing Rate. Competitive inhibition fitting utilizing Eq 4. The K_i_ for the addition of an I-40-dsDNA substrate was determined to be 200 nM ± 30 nM. The affinity per base pair can also be calculated utilizing Eq 5. Utilizing a binding footprint size of 24 bp, the K_i_ is calculated as 7 μM ± 1 μM. All reactions were performed a minimum of three times at 16°C. Error reported is the standard deviation for the replicates.
(TIF)
