# Supplementary material for: The Inhibitory Effect of Non-Substrate and Substrate DNA on the Ligation and Self-Adenylylation Reactions Catalyzed by T4 DNA Ligase
Source: PLoS One. 2016 Mar 8;11(3):e0150802. doi: 10.1371/journal.pone.0150802 (PMC4782999; doi:10.1371/journal.pone.0150802)
Supplement: S1 Table — The above table is a listing of all single- and double-stranded DNA substrates and their respective abbreviations used throughout the manuscript. (DOCX) [file pone.0150802.s001.docx]

|  | **Table S1: DNA Substrates** |  |
| --- | --- | --- |
| **Substrate Name** | **Substrate Sequences** |  |
| DNA-OH | 5’-GCGCACCCTTACCACCAAGACAGGATCGTCCTTGC–3’ |  |
| p-DNA | 5’-p-TGATCATGCATCGTTCCACTGTGTCCGCGACATCTACGTC-FAM-3’ |  |
| splint-DNA | 5’-GACGTAGATGTCGCGGACACAGTGGAACGATGCATGATCAGCAAGGACG  ATCCTGTCTTGGTGGTAAGGGTGCGC-3’ |  |
| DNA-OH-ddC | 5’-GCGCACCCTTACCACCAAGACAGGATCGTCCTTG**C**–3’ |  |
| p-DNA-noFAM/PO_4_ | 5’-TGATCATGCATCGTTCCACTGTGTCCGCGACATCTACGTC-3’ |  |
| I-75-dsDNA | 5’-AGAGAAGATGACTACTGAGTTGTGCGATCCCTGCACTTCAGCTAAGGAAG  CTACCAATATTTAGTTTCCGAGTCT-3'  5’-AGACTCGGAAACTAAATATTGGTAGCTTCCTTAGCTGAAGTGCAGGGATC  GCACAACTCAGTAGTCATCTTCTCT-3’ |  |
| I-75-ssDNA | 5’-AGAGAAGATGACTACTGAGTTGTGCGATCCCTGCACTTCAGCTAAGGAAG  CTACCAATATTTAGTTTCCGAGTCT-3' | |
| I-40-dsDNA | 5’-CACATATGAGAAGGTATTTGCCCGATAATCAATACTCCAG-3’  5’-CTGGAGTATTGATTATCGGGCAAATACCTTCTCATATGTG-3’ | |
