# Supplementary figures and images for: The Inhibitory Effect of Non-Substrate and Substrate DNA on the Ligation and Self-Adenylylation Reactions Catalyzed by T4 DNA Ligase
Source: PLoS One. 2016 Mar 8;11(3):e0150802. doi: 10.1371/journal.pone.0150802 (PMC4782999; doi:10.1371/journal.pone.0150802)

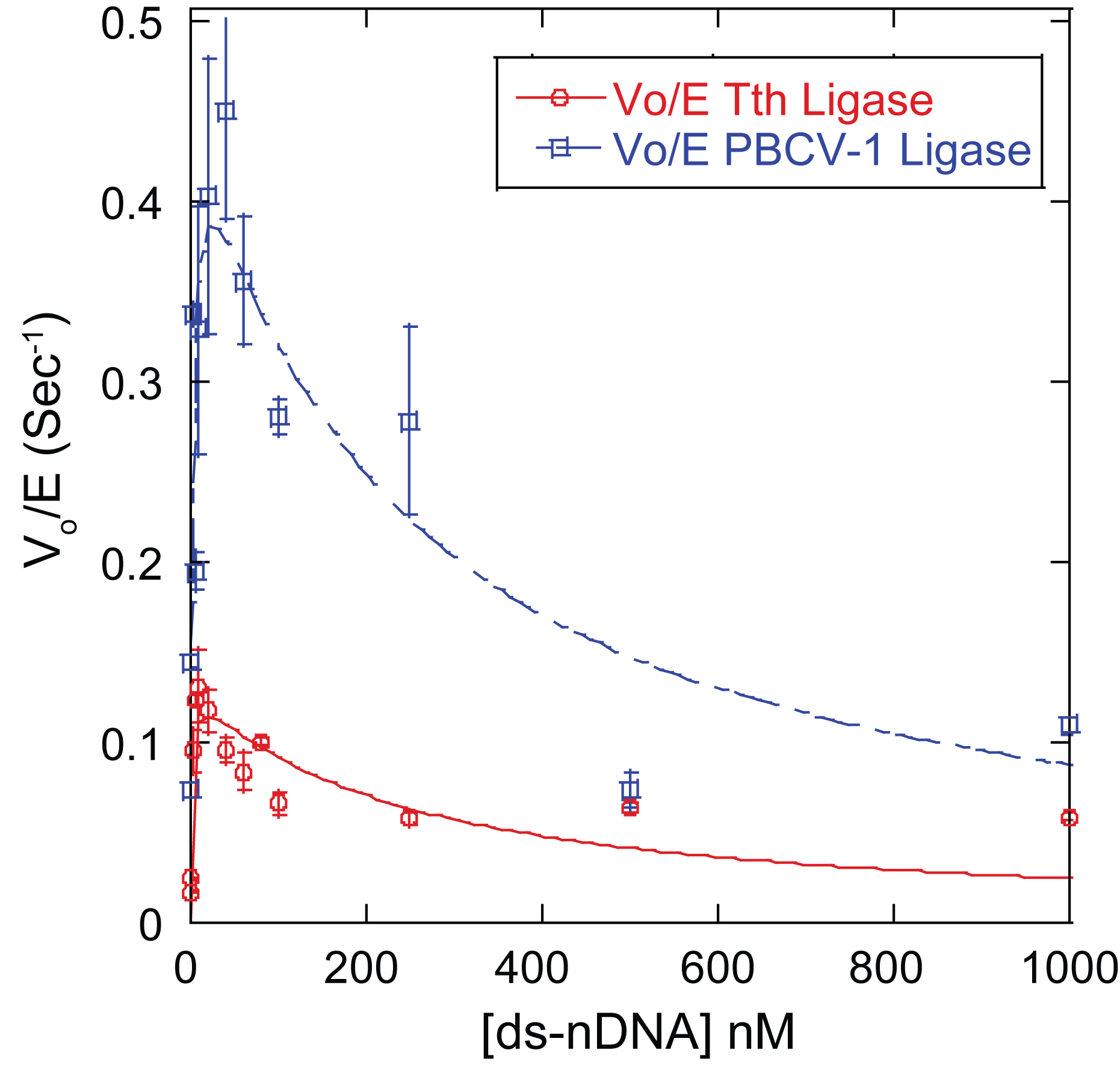

Supplement: S1 Fig — The data was obtained through titration of increasing concentrations of a 75mer-ds-nDNA substrate, reacted at 25°C (PBCV-1), and 55°C (Tth) to determine initial rates. Enzyme concentrations used were 25 pM– 100 pM for both PBCV-1 and Tth DNA ligase. The initial rates were plotted against their respective substrate concentrations and fit by a substrate inhibition model (Eq 2), where Km values of 1 nM ± 1 nM (PBCV-1), and 2.1 nM ± 0.9 nM (Tth) were determined. Ki values for the inhibition of each ligase were also determined 115 nM ± 60 nM (PBCV-1), and 200 nM ± 100 nM (Tth). All reactions were performed a minimum of three times. Error reported is the standard deviation for the replicates. (TIF) [file pone.0150802.s002.tif]

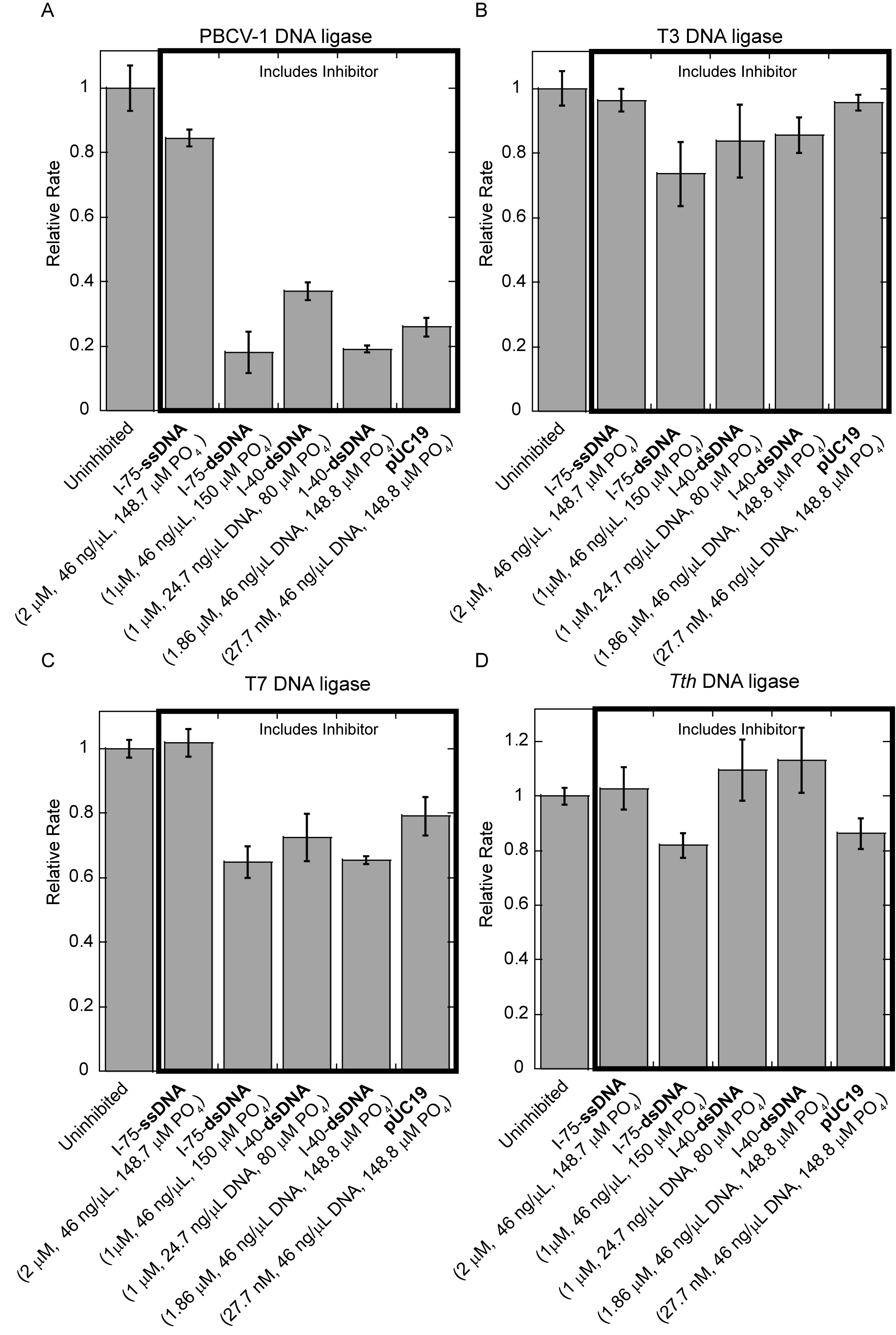

Supplement: S2 Fig — Various concentrations of dsDNA substrates were utilized as potential inhibitors of the steady state ligation reaction on 20 nM of the 75mer-ds-nDNA substrate. A. Reactions with 25 pM of PBCV-1 DNA ligase at 22°C. B. Reactions with 50 pM of T3 DNA ligase in T4 DNA ligase buffer at 25°C. C. Reaction with 50 pM of T7 DNA ligase in T4 DNA ligase buffer at 25°C. D. Reactions with 25 pM of Tth DNA ligase in Tth DNA ligase buffer at 55°C. All reactions were performed a minimum of three times. Error reported is the standard deviation for the replicates. (TIF) [file pone.0150802.s003.tif]

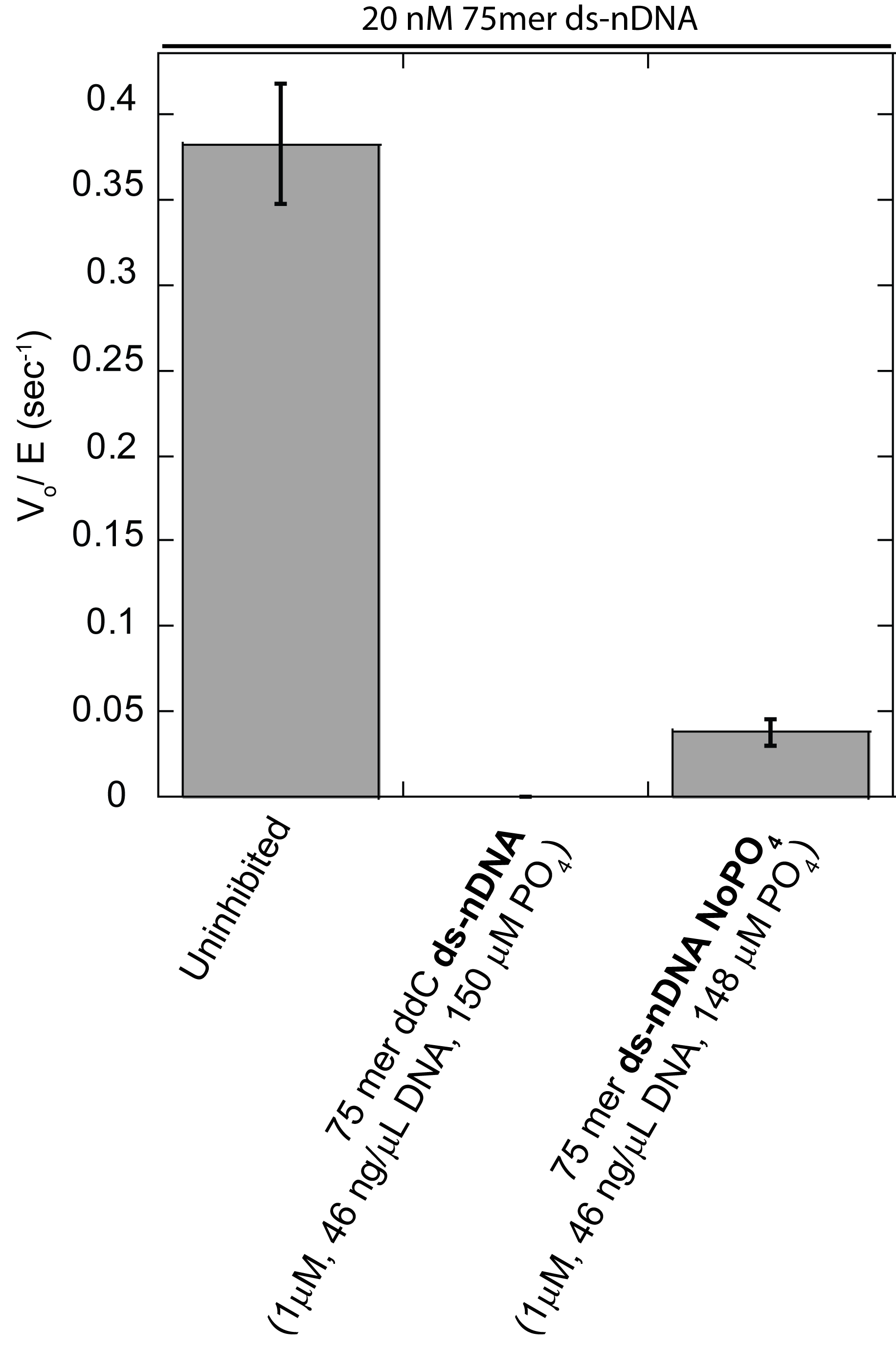

Supplement: S3 Fig — Effect of unlabeled dehydroxylated (ddC) and dephosphorylated (NoPO4) ds-nDNA substrates on the T4 DNA ligase steady state ligation reaction on 20 nM of the 75mer-ds-nDNA substrate. All reactions were performed in the presence of 25 pM of T4 DNA ligase at 16°C. All reactions were performed a minimum of three times. Error reported is the standard deviation for the replicates. (TIF) [file pone.0150802.s004.tif]

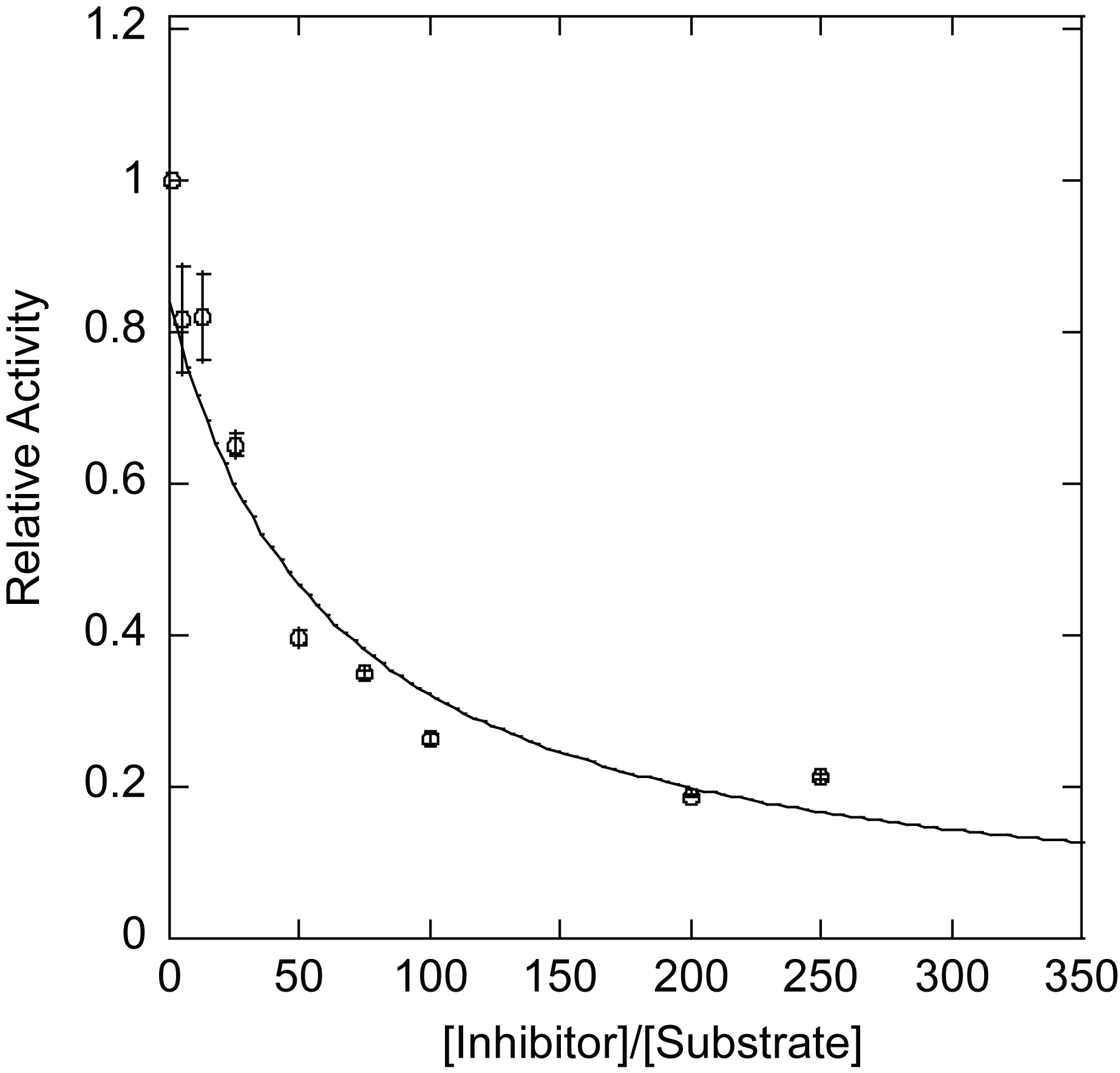

Supplement: S4 Fig — Competitive inhibition fitting utilizing Eq 4. The Ki for the addition of an I-40-dsDNA substrate was determined to be 200 nM ± 30 nM. The affinity per base pair can also be calculated utilizing Eq 5. Utilizing a binding footprint size of 24 bp, the Ki is calculated as 7 μM ± 1 μM. All reactions were performed a minimum of three times at 16°C. Error reported is the standard deviation for the replicates. (TIF) [file pone.0150802.s005.tif]
